# Peripheral Neuritis with Œdema

**Published:** 1904-08-27

**Authors:** C. O. Hawthorne

**Affiliations:** Physician to the Central London Ophthalmic Hospital; Assistant Physician to the North-West London and Royal Waterloo Hospitals.


### Hospital Clinics.

### PERIPHERAL NEURITIS WITH ŒDEMA:

A Clinical Lecture, by C. O. Hawthorne, M.D., M.R.C.P., Physician to the Central London Ophthalmic Hospital; Assistant Physician to the North-West London and Royal Waterloo Hospitals.

This patient—a man of 35 years—has come to seek medical advice because during the last fourteen days he has been troubled with pains in his lower limbs below the knees. These pains have not been very severe, but they have caused him a certain amount of annoyance in themselves, and, as he puts it, they have also "led" to weakness and to swelling of his feet and legs. The weakness has expressed itself in the form of occasional unsteadiness of gait, and once or twice his knees have suddenly given way and he has found himself sitting on the pavement. He appears to regard this development mainly from a jocular standpoint, and altogether speaks of his condition as though it had never given him much mental concern or anxiety. The most instructive method of studying the case will be, I think, to consider separately each of the three symptoms—pain, weakness, and swelling—of which the patient makes complaint, with a view to frame a diagnosis that will include these together with any other facts that may be discovered on physical examination. And in the first place it is evident that the swelling is a real thing, for there is manifest pitting on pressure over the dorsum of each foot and along the subcutaneous surface of each tibia; a like condition is also found over the spines of the sacral and lumbar vertebræ. The swelling is therefore due to œdematous effusion in these regions. It is advisable to emphasise this because frequently patients complain of swellings which have no objective existence, and therefore, unless examination reveals the fact, it is not safe to conclude that a complaint of this character necessarily means œdema. Presumably local irritation or some other cause may produce dilatation of the peripheral vessels in any part of the body, and this causes a subjective sense of swelling, though there is no œdema to physical examination. Hence painful parts may feel to be "swollen" to the patient, though there is no manifest change to the eye of the physician. But in the present case, as we have already seen, there is no room for uncertainty. In the patient's lower limbs and over the lower part of his back there undoubtedly is swelling, and this swelling is due to œdema. Now, it is obvious that this is a fact which claims an explanation, and perhaps, as a matter of experience, we expect that explanation in a male adult to be afforded most probably by disease of the heart or kidneys. An examination of the urine, however, fails to detect anything abnormal, and in particular all the tests for albumen give negative results. Similarly there is no evidence of valvular disease of the heart, and whilst, perhaps, a very critical examination may suggest a slight extension of the percussion dulness to the left, neither this nor the fact that the pulse is somewhat compressible implies a definite degree of cardiac dilatation likely to produce the œdema present in our patient. The condition of the lungs, also, as determined by physical examination, is normal, and therefore the œdema cannot be due to the obstructive influence of chronic bronchitis or emphysema on the right heart and the venous circulation. Examination of the abdomen, likewise, yields normal results, and hence there is nothing to suggest a tumour or other cause of pressure on the large veins as responsible for the œdematous state of the lower limbs and loins. In some cases œdema attends various anæmic states which produce grave general disability and cardiac weakness, but the general appearance of the present patient, apart from the negative results of physical examination, forbids such an explanation in the present instance. And such an exceptional cause of œdema as trichinosis need hardly be here discussed, for there has been none of the intestinal symptoms which attend that condition and neither widespread pain in the muscles nor fever. In short, in discussing the cause of the œdema we are compelled to exclude alike those general diseases—leucocythæmia, pernicious anæmia, malignant disease, etc.—which depreciate the quality of the blood and the force of the heart, as well as pressure on the large veins within the abdomen, and disease of the heart, lungs, and kidneys. Hence the attempt to utilise the fact of the œdema as a guiding line to a diagnosis has not proved very helpful; it is necessary therefore to turn our attention to other aspects of the case.

A second symptom of which the patient complains is weakness in his legs. Yet when the power of the various muscular groups is tested they appear fairly strong. The quadriceps and the hamstring muscles in each limb are certainly powerful, but there is possibly a certain degree of flabbiness and feebleness in the calf muscles and in the anterior tibial group. But even this is not very definite, though it is necessary to remember that as the condition is presumably bilateral, there is no opportunity for making specific comparisons between the two limbs, and therefore a relatively slight deficiency may fail to be confidently appreciated. When, however, the patient is made to walk across the ward there is no doubt of some disability in the motor apparatus. He walks, as you observe, with his feet wide apart, and his gait is deficient in confidence—he cannot, as he says, "trust his legs." Further, he is unable to stand with his feet close together whilst his eyes are closed. It is therefore obvious that whether there is or is not defective power, ataxia is definitely present. The patient is unable completely to co-ordinate the several parts of his muscular apparatus for such purposes as walking and the maintenance of equilibrium. Another very important fact that appears on examination is complete absence of each knee jerk. That is a conclusion which must never be assumed without careful examination. It is not sufficient merely to have the knees crossed and to strike the patella tendon. If this is followed by the development of the knee-jerk well and good; but if not, more severe tests must be applied. Hence we make this patient practise "re-inforcement" by hooking the fingers of one hand within those of the other and exercising traction so as to distract his attention from the lower limbs and to heighten as it were the tension of the whole nervo-muscular mechanism, and we deliver the stroke over the tendon not through the clothing but directly on to the naked skin. But tested in this and in other ways no response in the shape of the knee jerk can be elicited. Now a combination of ataxia in the lower limbs with absence of the knee jerks cannot, in a man approaching middle-life, fail to suggest the diagnosis of locomotor ataxia. Further, there is in the present case a complaint of pain in the lower limbs, though this has not the shooting or lightning-like character which is the usual kind of pain in this disease. And, in addition, the state of the sensory functions of the skin below the knee might quite justifiably be quoted in support of the diagnosis of locomotor ataxia. This latter condition is sufficiently interesting to be noted in some detail.

It is in the first place obvious that there is marked deficiency of the capacity to appreciate painful stimuli, for when a needle is run through a fold of skin picked up from the surface of either leg the patient neither shrinks nor complains of pain. There is also some deficiency of the thermal sense, as is shown by the want of confidence in distinguishing between a hot and a cold sponge. But tactile sensation, though perhaps a little dulled, is much less depreciated than the painful and thermal forms of sensation. Such a condition of the sensory functions is not, perhaps, particularly suggestive of locomotor ataxia, but it is by no means out of harmony with that diagnosis. The disease in which this loss of the sense of pain and temperature with retention of tactile sense is most conspicuously presented is syringomyelia. But in this condition there is a gradually increasing muscular atrophy, usually affecting the upper limbs; the course of this disease also is a very chronic one, and the knee-jerks are apt to be exaggerated rather than lost. Hence syringomyelia hardly needs to be discussed as a possible diagnosis in the present case. The disturbance of the sensory functions in the parts below the knees may, however, fairly enough be combined with the character of the gait and the absence of the knee-jerks in support of the diagnosis of locomotor ataxia. But can the œdema be explained by this diagnosis? This is perhaps just possible, but that is all that can be said for it. Sometimes locomotor ataxia, as is well known, leads to swelling and other changes in the joints as a result of some disturbance of the trophic nerve mechanism, and with this there may be more or less subcutaneous œdema. Hence it is just conceivable that the latter might occur apart from the joint affections, and the statement has been made that this does occasionally happen. But it is certainly so exceptional an event that, unless the diagnosis is very secure and all other possible interpretations can be confidently excluded, locomotor ataxia cannot safely be considered as a sufficient explanation of the presence of œdema. Now we have taken pains here to exclude the usual causes of œdema, but if we desire to uphold the diagnosis above suggested it will be wise to endeavour to obtain, if possible, other facts in its support. But in this quest we do not meet with much encouragement. Thus, there are none of the ocular symptoms of locomotor ataxia. The pupils have a perfectly good light-response, the optic discs are not unduly white, the visual acuity is good, and the fields of vision show no evidence of the peripheral contraction which is often an early sign of optic atrophy. Again, the patient denies anything like the girdle-sensation, any disturbance of the functions of the bladder, or any modification of the sexual function; and on testing the trunk there is not at any level that zone of deficient sensation which may sometimes be observed comparatively early in the disease. If, therefore, the diagnosis of locomotor ataxia is to be maintained it must find its support solely in the character of the gait, the absence of the knee-jerks, and the abnormal sensory condition. These, speaking generally, may be ample to support such a diagnosis, but in the present instance the absence of confirmatory facts and the presence of œdema compel us to scrutinise them with an extra degree of care, and also to inquire whether both they and the œdema may not be more safely covered by some alternative explanation. Now, regarding the loss of the knee-jerks and the character and degree of the anæsthesia there is nothing more to be said; they are without question well within the province of locomotor ataxia. The gait is hardly defined with equal confidence. There is, it is true, an ataxic-like character about it, especially when the patient is asked to walk along a narrow line, but at the same time it scarcely has the "stamping" element seen in locomotor ataxia. Further, there is probably some degree of weakness in the muscles below the knee, and neither this nor the pain of which the patient complains is suggestive of that disease. Altogether, therefore, there is room for hesitation before concluding that the present case is one of locomotor ataxia attended by a very unusual complication in the shape of subcutaneous œdema. What suggestion then can be set in opposition to this diagnosis? Now it may be usefully remembered that, whilst evidences of nervous disease having a bilateral distribution suggest in the first place a lesion of the spinal cord, because here the two halves of the central nervous system are in close anatomical association, there are certain other possibilities to be considered. And one of these is that the lesion producing the bilateral manifestations may be, not in the cord, but in the peripheral nerves. Grant the introduction into the blood of some poison capable of producing prejudicial changes in the peripheral nerve-endings, say in the skin and muscles, and there must inevitably result bilateral symptoms of sensory and motor defect. Will, then, a multiple symmetrical peripheral neuritis fit the facts of the present case? Obviously such a diagnosis will explain the absence of the knee-jerks, because it involves a breach of the reflex arc upon which the fact of the knee-jerk depends, and the free and ready transmission of nervous impulses between the nerve-centres of the spinal cord on the one hand and the muscles on the other is therefore no longer possible. Peripheral neuritis will also account for the sensory defect and for the pain quite as well, if indeed not more satisfactorily, than the alternative diagnosis of locomotor ataxia. There is also in the case another sensory condition which peripheral neuritis readily explains—namely, some degree of muscular tenderness. It is by no means as marked here as in many cases, but quite beyond question a fairly firm grasp of the calf-muscles causes the patient an abnormal degree of discomfort. The absence of the knee-jerks and the cutaneous anæsthesia, then, adapt themselves equally to either of the two diagnoses now under discussion, whilst the character of the pain and the muscular hyperæsthesia—the latter especially—favour the diagnosis of peripheral neuritis. Considering next the gait, it is manifest that peripheral neuritis may also explain the defect in this direction. Between the spinal centres and the muscles there is, it must be remembered, a double set of communicating nerve-fibres. Along one pass efferent impressions which stimulate the muscles to contraction. The others convey afferent impressions from the muscles to the nerve-centres and thus, as it were, by keeping the centres informed of the state and condition of the muscular fibres, enable the centres to adjust their activity to the degree required for the contraction of the several muscles engaged in the performance of various complex actions, such as walking, balancing, etc. Now a lesion which interferes with the endings in the muscle of nerve-fibres carrying impulses from the spinal centres to the muscles must necessarily cause a greater or less degree of muscular weakness—the impulses cannot fully reach and successfully stimulate the muscle. On the other hand, a lesion involving the peripheral nerve-endings through which impressions travel to the spinal cord must necessarily mean that the spinal centres are badly informed regarding the condition of the muscles, and hence they will fail to adapt their activity to the degree of muscular action needed for specific ends. In the first case there is muscular weakness or paresis; in the second there is ataxia. A combination of these two symptoms, therefore, is specially suggestive of peripheral neuritis, and it is this combination which exists in the present case. The more usual arrangement is for the paretic condition to be in excess, and to be attended with muscular atrophy. But one only has to suppose that in the present instance it is the second group of fibres (as above defined) that have mainly suffered, to have an explanation of the predominating ataxia, of the less marked evidence of muscular weakness and flabbiness, of the pain from which the patient suffers, and of the muscular hyperæsthesia. A review of all these facts would thus seem to justify the suggestion that they find their explanation rather in a diagnosis of peripheral neuritis than in that of locomotor ataxia. There remains, however, the question of œdema. This may, as we have seen, be just possibly brought within the diagnosis of locomotor ataxia. Is it or is it not more easily accommodated in that of peripheral neuritis? There is little difficulty in answering this question in the affirmative. Indeed, in one disease distinguished by a peripheral neuritis, namely beri-beri, œdema is a common and prominent feature. Another illustration may perhaps be found in chronic arsenical poisoning, in which both peripheral neuritis and œdema (usually of the eyelids) may be seen. Alcohol in this respect stands in much the same position. Probably the explanation of the œdema is to be found in a neuritis of vaso-motor nerves exactly comparable to the neuritis which in motor nerves produces muscular weakness, atrophy, and ataxia, and in the sensory nerves pain and other disorders of sensation. It is certainly highly improbable that our patient is suffering from beri-beri, if only for the reason that he has never been out of this country. Equally arsenical poisoning, in the absence of any history which would suggest it, of undue pigmentation of the skin, and of keratosis of the soles or palms, need hardly be considered. But the possibility of alcohol cannot lightly be dismissed. The patient is a commercial traveller, and he admits he is in the habit of taking frequent "nips" of whiskey, and that sometimes he has "a drop too much" both of beer and whiskey. Without any departure from charity, therefore, we may assume that for some considerable time his habits have favoured the production of peripheral neuritis and that the history of the case inclines so far to that diagnosis. Diphtheria, diabetes, lead poisoning are other causes of peripheral neuritis, but they can all be excluded from the present case. Thus, when all the facts are reviewed, there can be little hesitation in departing from the diagnosis of locomotor ataxia and adopting that of peripheral neuritis. There is not the degree of muscular atrophy often seen in that disease, and the motor defect is less that of weakness than of ataxia. But as the case is an early one the absence of muscular wasting does not militate against the diagnosis, and the predominance of ataxia can be explained in the manner already suggested; the diagnosis can also be supported by the analogy of what is often found in diphtheritic neuritis. The neuritis in our patient apparently involves nerve endings in the muscles, the skin, and in vaso-motor tracts, and so produces disturbances in the motor, the sensory, and the vaso-motor functions. It is to the latter that the œdema is in all probability to be attributed.

The diagnosis of peripheral neuritis enables us to give a much more hopeful prognosis than had we been compelled to regard the case as one of locomotor ataxia, and the early stage of the case strengthens this impression. By cutting off the supplies of alcohol we are able to remove the cause of the disease, and this, combined with rest, will doubtless be followed by a gradual return of the damaged nerves to a normal condition. The same influences will tend to get rid of the œdema, and as soon as the muscular tenderness has subsided—and almost at once, indeed, as this is relatively slight—strychnine and the adoption of massage may be commenced with a practical certainty that the patient will ere long be restored to good health.

